# Brain activity sustaining the modulation of pain by empathetic comments

**DOI:** 10.1038/s41598-019-44879-9

**Published:** 2019-06-10

**Authors:** C. Fauchon, I. Faillenot, C. Quesada, D. Meunier, F. Chouchou, L. Garcia-Larrea, R. Peyron

**Affiliations:** 1 Central Integration of Pain, Lyon Neuroscience Research Center, Inserm U1028, UCB Lyon1 & UJM, F-42023 Saint-Etienne, France; 2 Department of Neurology & Pain Center, CHU de Saint-Etienne, F-42055 Saint-Etienne, France; 3 Aix Marseille Univ, CNRS, INT, Inst Neurosci Timone, Marseille, France

**Keywords:** Sensory processing, Empathy

## Abstract

Empathetic verbal feedback from others has been shown to alleviate the intensity of experimental pain. To investigate the brain changes associated with this effect, we conducted 3T-fMRI measurements in 30 healthy subjects who received painful thermal stimuli on their left hand while overhearing empathetic, neutral or unempathetic comments, supposedly made by experimenters, via headsets. Only the empathetic comments significantly reduced pain intensity ratings. A whole-brain BOLD analysis revealed that both Empathetic and Unempathetic conditions significantly increased the activation of the right anterior insular and posterior parietal cortices to pain stimuli, while activations in the posterior cingulate cortex and precuneus (PCC/Prec) were significantly stronger during Empathetic compared to Unempathetic condition. BOLD activity increased in the DLPFC in the Empathetic condition and decreased in the PCC/Prec and vmPFC in the Unempathetic condition. In the Empathetic condition only, functional connectivity increased significantly between the vmPFC and the insular cortex. These results suggest that modulation of pain perception by empathetic feedback involves a set of high-order brain regions associated with autobiographical memories and self-awareness, and relies on interactions between such supra-modal structures and key nodes of the pain system.

## Introduction

Humans have the capacity to estimate each others’ pain and to provide adapted care in order to reduce it. It is largely assumed that empathetic skills are crucial for caregivers involved in pain management^1–4^; consequently, educational programs and theories have emphasized the positive role of empathy to reduce pain intensity. It is also widely assumed that if caregivers lack empathy, they will underestimate pain intensity in their patients and this unempathetic attitude can negatively influence pain intensity perception. In a recent study, we addressed this issue by creating a dedicated setup mimicking a medical environment where people enduring painful stimuli received empathetic or unempathetic comments from others. Positive (empathetic) feedback was able to reduce pain intensity perception, in agreement with previous theoretical models^5,6^ and experimental studies^7^, while negative (unempathetic) feedback did not induce consistent changes in pain intensity reports^8^.

Here, we transposed the experimental setup employed in our preceding behavioral study^8^ to functional magnetic resonance imaging (fMRI). To heighten the realism, healthy subjects placed in the scanner received thermal noxious stimuli while overhearing positive, neutral or negative comments about them, made by the experimenters, via an ‘inadvertently’ switched-on communication device. To our knowledge, no neuroscientific study has experimentally investigated the neural mechanisms mediating the effects of empathetic or unempathetic feedback on pain. BOLD (Blood Oxygen Level Dependent) activity and functional connectivity (FC) changes related to pain intensity perception were compared according to these three experimental conditions. Our objective was to investigate how empathetic or unempathetic comments could interact with the brain structures underlying the assessment of pain intensity.

The current concepts about cortical pain integration consider that there is, in the conscious appraisal of pain, a need to integrate sensory encoding with stimulus salience, executive control, memory encoding and self-awareness^9–13^. Thus, the neural mechanism of pain can be conceptualized as a system composed of multiple functional modules of brain regions interacting together to achieve different subgoals^9,11,14^. A ‘nociceptive subnetwork’ responsible for processing the sensory aspect of pain includes regions receiving input from the ascending spinothalamic tract (e.g., posterior operculo-insular regions and S1). A further set of subnetworks recruited by nociceptive input can support the ‘salience’ attributes of noxious stimuli, trigger top-down cognitive controls, and ensure the conscious evaluation of pain intensity (e.g., anterior insula, mid cingulate cortex, dorsolateral prefrontal and posterior parietal cortices). Affective-cognitive processes including, expectations, beliefs, contextual factors, and self-awareness can still modulate the conscious experience of pain via activity in supramodal regions with widespread cortical projections (e.g., ALPFC, posterior parietal cortex, precuneus, posterior cingulate cortex). Pain modulation by empathy or pleasant/unpleasant contexts tends to change activity in higher order executive and salience networks^15,16^ and sometimes secondarily in sensory cortices^17^. A few studies have examined the brain mechanisms associated with receiving social support^18^ from the participant’s romantic partner (i.e., the effect of viewing pictures^19^, holding the hand^20^ or the physical presence in the room^21^) during a pain experience. Brain activity associated with pain reduction in these conditions was found in areas responding to “safety” (i.e., the ventromedial prefrontal cortex and the posterior cingulate cortex)^18^ and self-regulatory processes (i.e., dorsolateral prefrontal cortex)^20^. A recent work by Hein and colleagues^22^ pointed out that pain relief provided by an outgroup member decreased right anterior insula activity.

Since the pain experience results from the interaction of sensory brain areas with higher-order brain networks^9,10,23^, we postulated that empathetic feedback would modulate pain-related responses through changes in these high-level brain areas associated with inferring and representing states of self and other awareness^24^. We hypothesised that brain areas (i) responding to “safety” (i.e., the ventromedial prefrontal cortex and the posterior cingulate cortex); (ii) implicated in self-regulatory processes (i.e., dorsolateral prefrontal cortex) and (iii) contextual processing (i.e., anterior insula) would be involved in the cerebral mechanisms related to the reappraisal of pain perception by others’ empathetic comments.

## Results

### Brain responses to verbal and pain thermal stimuli over the entire experiment

**Figure 1:**
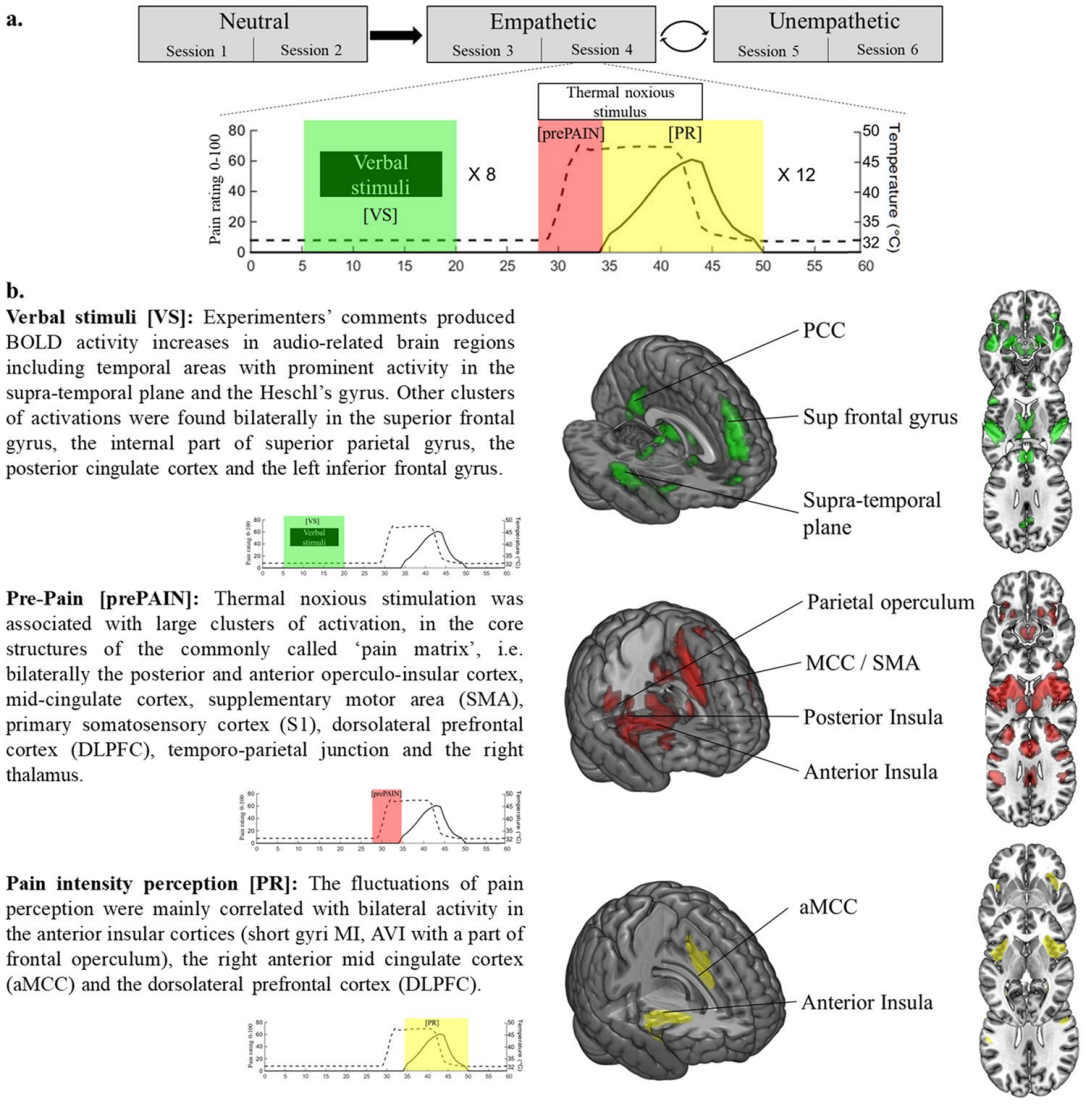
Brain activations related-to pain stimulations and audio comments over the whole experiment. (**a**) The MRI task was split in six sessions of 8 min (i.e., two successive sessions for each experimental condition). Empathetic and Unempathetic conditions were randomly assigned between subjects. Each session was composed of 12 thermal noxious stimuli alternating with 8 verbal stimuli. For fMRI analysis, three regressors were considered: verbal stimuli (green; [VS]), noxious thermal stimulation (red; [prePAIN]) and ratings of pain intensity perception on a scale of 0–100, where 0 is no pain and 100 is maximum imaginable pain (yellow; [PR]). (**b**) [VS] and [prePAIN] are associated with brain activity in the core areas of the auditory and pain networks. Pain intensity ratings [PR] are mainly associated with brain activity changes in the anterior mid-cingulate (aMCC), anterior insula, and dorsolateral prefrontal cortex (DLPFC). Thresholded at *p* < 0.01, FWE corrected.

Brain activity related to experimenters’ comments, thermal noxious stimuli, and fluctuation of pain intensity perception were examined for the whole fMRI experiment (Fig. 1a) across all the conditions, and thresholded at *p* < 0.01 corrected (family-wise error, FWE voxel level). Verbal stimuli [VS] were associated with brain activity in the auditory network. Thermal noxious stimuli [prePAIN] activated cortical areas commonly related to nociceptive stimuli (i.e., the posterior and anterior operculo-insular cortex, mid-cingulate cortex, SMA, primary motor and somatosensory cortex) while conscious pain intensity ratings [PR] were mainly associated with brain activity changes in the anterior mid-cingulate (aMCC), anterior insula and dorsolateral prefrontal cortex (DLPFC). More details are reported in Fig. 1b. For the full list of activated brain regions see Supplementary Table 1.

### Effect of empathetic conditions on behavioural scores and pain brain activity

#### Pain perception scores

**Figure 2:**
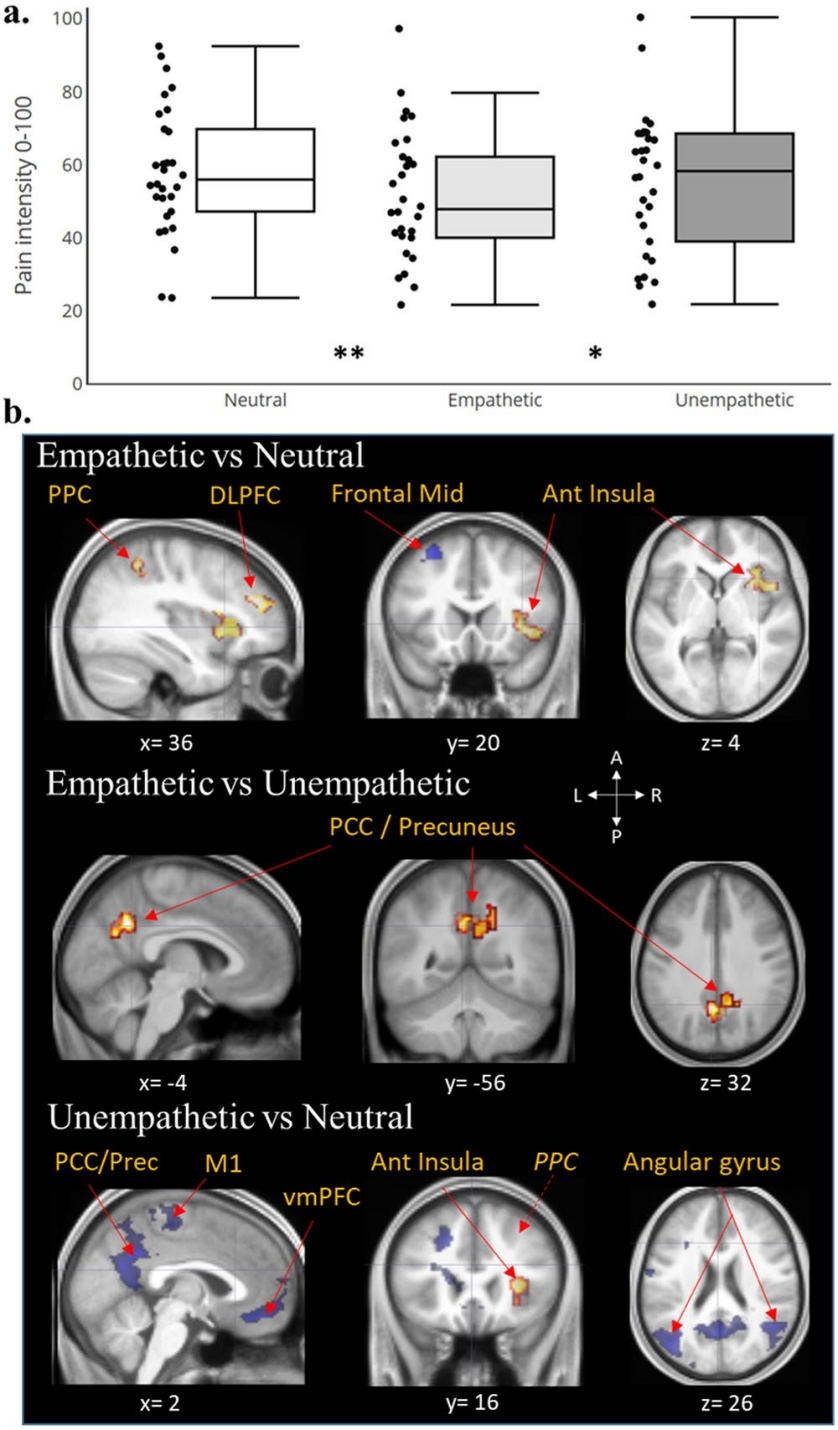
Brain correlates associated to experimental conditions effects on pain intensity perception. (**a**) Box plots (with median and quartile) and scatter plots of individual pain scores related to experimental conditions. Empathetic condition reduced significantly pain ratings as compared to Neutral (**p < 0.01) and Unempathetic conditions (*p < 0.05). (**b**) Activations (red: E or U > N) and deactivations (blue: N > E or U) superimposed on the anatomical scan averaged across all subjects. Empathetic and Unempathetic feedbacks from others both activated the right anterior insular cortex and the right posterior parietal cortex (PPC) compared to the Neutral condition. In addition, empathetic comments increased activity in the right dorso-lateral prefrontal cortex (DLPFC) and decreased the middle frontal gyrus whereas an Unempathetic situation induced mainly a deactivation in the default mode network structures. Only, the activity of the Posterior Cingulate Cortex/Precuneus (PCC/Prec) distinguished between these two opposite contexts (E > U). Statistical maps are thresholded at FWE-corrected cluster-based *p* < 0.05 after voxel threshold at *p* < 0.001.

Pain ratings varied significantly according to experimental conditions (*F*(2,28) = 3.89; *p* = 0.026) without interaction between condition and order of presentation (*F*(2,28) = 0.59; *ns*). Post-hoc tests showed a significant decrease of pain ratings in the Empathetic condition (PRmax = 50.41) as compared to both the Neutral (PRmax = 58.10; −13% *p* = 0.007) and Unempathetic conditions (PRmax = 55.23; −9% *p* = 0.047). No difference was found between Unempathetic and Neutral conditions (*p* = 0.23). Figure 2a depicts these findings. The latency from the onset of the thermal noxious stimuli to the onset of pain ratings was also computed. No significant influence of experimental conditions (*F*(2,28) = 2.50; *p* = 0.08) on subject’s pain perception response latency was found.

### Brain activity related to experimental conditions during pain perception

#### Empathetic feedback

During the Empathetic condition, BOLD activity associated with pain intensity increased significantly in the: right anterior insular cortex, right posterior parietal cortex (PPC) and DLPFC, while it decreased significantly in the left middle frontal gyrus in the Empathetic condition as compared to Neutral control (*t*(30) > 5.66). Compared to the Unempathetic condition, pain intensity ratings during the Empathetic condition were specifically associated with bilateral activity increases in the ventral posterior cingulate cortex (PCC) and the precuneus (Prec) including a slight overlap with the inferior parietal lobule (IPL) and the temporoparietal junction (TPJ) (*t*(30) > 3.75; Fig. 2b).

#### Unempathetic feedback

During the Unempathetic condition, BOLD activity associated with pain intensity increased significantly in the right: anterior insula (short gyrus), the IPL part of the PPC (*t*(30) > 5.02) and decreased bilaterally in the angular gyrus, precuneus, posterior cingulate cortex, ventro medial prefrontal cortex (orbital and subgenual), hippocampus, the middle part of temporal gyrus and the right primary motor cortex (*t*(30) > 5.63; Fig. 2b), relative to the Neutral condition. See Supplementary Table 2 for more details on brain activations’ localisations.

### Modulation of FC related to pain in experimental conditions

**Figure 3:**
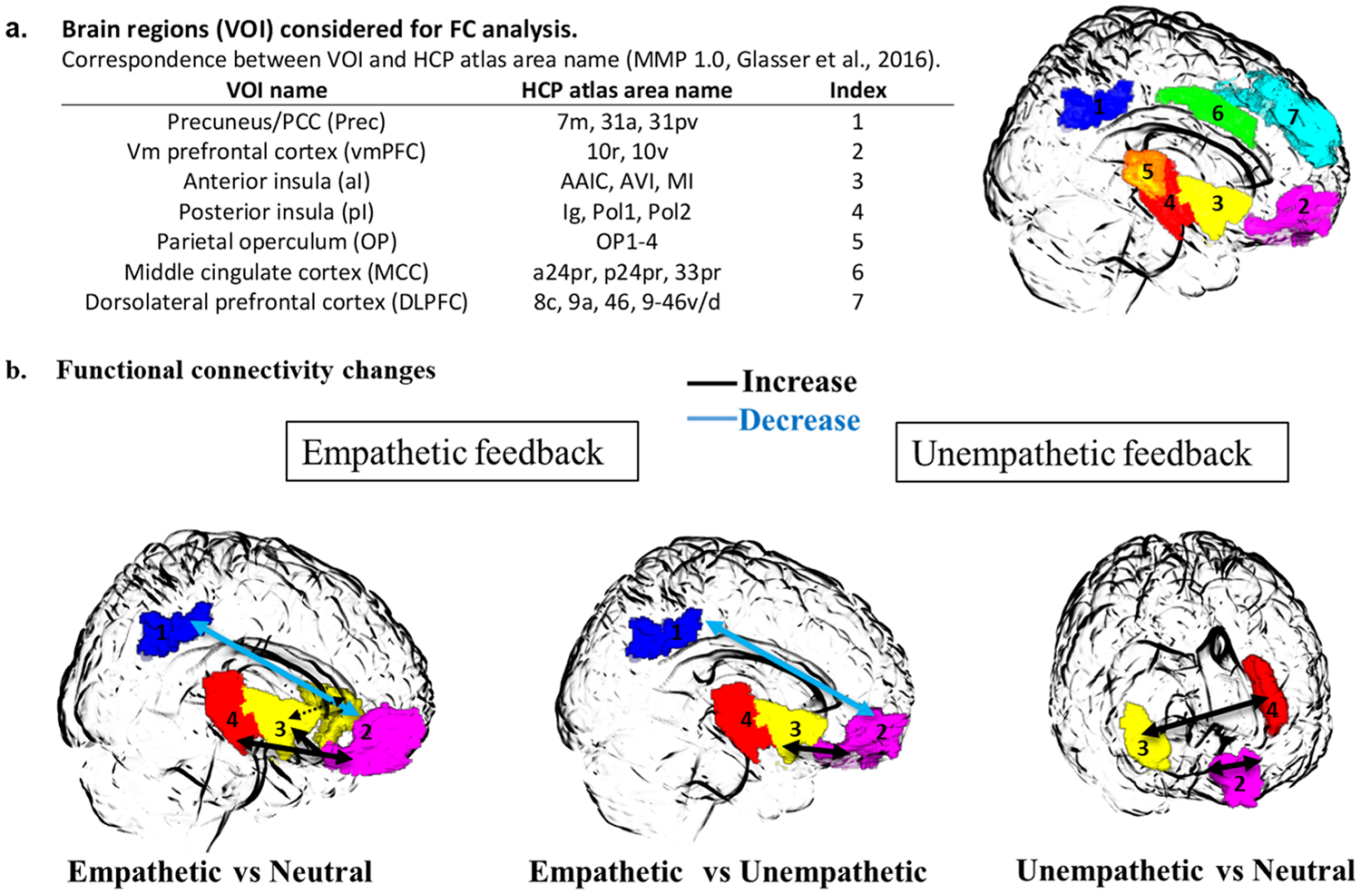
Functional connectivity (FC) changes according to experimental conditions during pain stimulation. (**a**) List of brain regions considered for FC comparison analysis projected on a glass brain with HCP correspondences for pain-stimulation based pairwise correlation matrix (14 × 14). (**b**) The three 3D-glass brains represent the FC changes for the paired comparisons of experimental conditions. Empathetic feedback induced significant increase of connectivity between vmPFC and both posterior and anterior insula, between both anterior insular cortices (dotted line) and a FC decrease between vmPFC and PCC/Prec relative to the Neutral and Unempathetic conditions. Only FC changes between vmPFC and pI was not significant as compared to the Unempathetic condition. Unempathetic feedbacks induced only inter-hemispheric increases of FC between both vmPFC, and between aI and pI (*p* < 0.05, FP-corrected).

The main activation clusters that differentiated the Experimental conditions (Fig. 2b) were the anterior insula (aI), the posterior cingulate cortex/Precuneus (PCC/Prec), the dorsolateral prefrontal cortex (DLPFC) and the medial prefrontal cortex (vmPFC) including the subgenual part of the anterior cingulate. We also included brain structures associated with pain stimuli that corresponded mainly in our experiment to the mid-cingulate cortex (MCC), the parietal operculum (OP) and the posterior insula (pI). The 7 brain regions (PO, pI, aI, MCC, PCC/Prec, vmPFC, DLPFC) considered bilaterally for FC analysis are depicted on Fig. 3a.

#### Empathetic feedback

Most of the changes in FC concerned the comparisons between Empathetic minus Neutral conditions: In the Empathetic condition time-series correlations increased between right pI and bilateral vmPFC areas, between both anterior insular cortices, between right anterior insula and right vmPFC (*t*(30) > 2.90, *p* < 0.007). The FC between left vmPFC and right PCC/Prec (*t*(30) = −3.27, *p* = 0.003; Fig. 3b).

FC increased significantly in empathetic conditions between left anterior insula and right vmPFC and decreased between right PCC/Prec and left vmPFC (*t*(30) = 3.51, *p* < 0.001) as compared to the Unempathetic conditions.

#### Unempathetic feedback

Comparing the Unempathetic and Neutral conditions, time-series correlation increased significantly between bilateral left and right vmPFC and between right anterior insula and left posterior insula (*t*(30) > 2.85, *p* < 0.008; Fig. 3b).

## Discussion

On behavioral grounds, this study confirms previous results indicating that receiving empathetic feedback from others can decrease the perceived intensity of pain, whereas unempathetic feedback appeared less powerful to enhance pain ratings, at least in our experimental conditions. The average magnitude of pain relief (−12%) is also consistent with results reported in our previous behavioral study^8^ and gives further evidence for the beneficial effects of empathetic support during acute pain. Furthermore, the present study provides some insight on the neural mechanisms by which empathetic support may modulate pain perception.

The key cerebral areas of the pain network^10,12,25,26^ and auditory network^27^ were vigorously activated by, respectively, the thermal noxious stimuli and the verbal comments (Fig. 1b & Supplementary Table 1). The introduction of two separate regressors, one corresponding to the period between stimulus onset and pain threshold [prePAIN], and the other restricted to the window where subjective pain ratings were produced [PR], permitted identification of brain regions associated with the initial reception of the stimulus, the subjective pain perception, or both (Fig. 1b). Thus, areas receiving spinothalamic input such as the posterior operculo-insular cortex and posterior MCC were active during the first portion of the evaluation ([prePAIN], stimulus detection) but were not significantly activated in connection with the regressor strictly associated with the conscious assessment of pain [PR], which in turn yielded significant bilateral activation of a smaller network comprising the anterior insula (a-Ins), the dorsolateral and anterolateral PFC, and the anterior mid-cingulate cortex (aMCC). This result is consistent with previous studies^28–30^ indicating that the coding of the conscious sensation of pain may be dissociated from the initial “nociceptive network” composed of sensory and motor/premotor areas^9,10,31^. This is highlighted by the results of contrast: [prePAIN] > [PR], and shown in Supplementary Fig. 4. These ‘later acting’ cerebral regions are not only associated with working memory, but also appear to be involved in the cognitive act of rating pain magnitude, and may participate in pain control^32^. The neural mechanism underlying conscious pain can be conceptualized as a fluid system composed of several interacting networks with different temporal dynamics^9–11,23^. The a-Ins, DLPFC, aMCC and PPC are activated later than the first somatosensory network, which receives the noxious inputs from the periphery^14^. These brain regions also support the transition to conscious awareness, transforming nociception into a pain sensation that may be influenced by multiple cognitive modulations (e.g., attentional, anticipative, and contextual-such as receiving empathetic support) from supramodal structures outside this network^9^.

The activity of the right a-Ins and right PPC (including the inferior parietal lobe and temporo-parietal junction) increased during both the Empathetic and Unempathetic conditions. Hence, these structures appeared to be globally influenced by the emotional context and did not dissociate positive from negative feedback, in accordance with their participation to a non-specific ‘salience’ network^33–35^. The engagement of the PPC may support the subject’s attentional shift to relevant (positive or negative) feedbacks^36^. The aI is a multimodal cortex^37^, generally recruited by components of the environment that are behaviorally relevant. Accordingly, neuroimaging studies have repeatedly shown enhancement of aI activity by the presentation of emotional stimuli (e.g. pictures, sounds, etc.) either positive^38–40^ or negative^41,42^. Although, this region is considered to play an important role in pain processing^43^, its activity is certainly not specific to pain^10,35,37^, and in our experiments did not reflect the modulation of pain scores by empathetic comments. Unchanged aI activity despite profound changes in subjective pain rating was previously reported during tasks involving highly emotional contexts; e.g., where pain rating was changed by religious beliefs^44^ or through observation of pain in others (compassional hyperalgesia)^16^.

In our experiments, the enhanced BOLD activity in the PCC/Precuneus, vmPFC and DLPFC was related to a decrease of pain intensity ratings in the Empathetic condition, whereas activities in the same regions decreased in the Unempathetic condition, as compared to Neutral control. These structures, in particular the two former, have been associated with different functions involved in the processing of self-oriented attention and autobiographical recall^45–47^, and are also known to play a central role in our ability to represent others’ emotional state^48–51^ (e.g., perspective taking and “theory of mind”). In addition, engagement of PCC/Prec and vmPFC has previously been shown in subjects receiving support from others during negative situations such as stress, threat or pain^18,20^. The opposite behavior of these regions in the Empathetic and Unempathetic conditions may suggest a differential integration of the experimenters’ comments, according to their valence^52^, in processes associated with the self and participants’ memories. This differential activity in fronto-parietal brain structures appears to be involved to the reappraisal of pain experience.

One intriguing, albeit robust, result was the decrease of functional connectivity (FC) between posterior and anterior midline regions (PCC/Prec and vmPFC) during pain stimulation in the Empathetic condition only. These are key nodes of the brain ‘default mode network’ (DMN) and recent work has described the importance of dynamic communication amongst DMN subnetworks in the pain experience^23^. Disengagement of attention from pain (i.e., mind-wandering) has also been associated with downplay of pain-induced DMN deactivation, that may prompt the engagement of pain control systems^53^. On the contrary, enhanced functional connectivity between vmPFC and PCC/Prec was observed in chronic pain patients, and was correlated with the degree to which patients ruminated^54^. One hypothesis could be that, in our experiments, empathetic feedback facilitated the detachment of subjects’ attention from thermal painful stimulation, reducing pain ratings to the same degree distraction does (around −10%)^55,56^.

A concomitant enhancement of FC was observed during the Empathetic condition between the above midline structures (PCC/Prec, vmPFC) and the insular cortex (both anterior and posterior parts) contralateral to the stimulation side, whereas FC increase was limited to inter-hemispheric connections in the Unempathetic condition. Although any concrete explanation can only be speculative at this point, these results support the notion that changes in pain-related behavioral responses may be under the influence of top-down control in synchrony between poly-supramodal areas which instantiate the “self” (i.e., PCC/Prec, DLPFC, vmPFC) and insular cortices (i.e., a-Ins and p-Ins).

A number of limitations should be taken into consideration when interpreting our results. First of all, participants passively listened to the experimenters’ conversation while lying inside the MRI scanner and did not participate in real exchanges. Our results cannot therefore provide a full picture of the neural processes involved during patient-caregiver interactions. Also, the lack of behavioural metrics of attentional focus and mood ratings during the different conditions, due to the experiment and the fMRI constraints, restricts the interpretation of some of the neural mechanisms proposed here. However, the results are consistent with changes in mood found in previous psychophysical studies^8,57^. Interestingly, such changes appeared to be especially linked to the empathetic comments which were unanimously considered as “positive” and “pleasant” on debriefing^8^, whereas less than one third of subjects considered unempathetic comments as”harmful” or “injurious”. It is therefore likely that the brain activity changes observed are related to the subjective impression of mood change. However, in the absence of any quantitative evaluation it was not possible to assess this relationship. Our hypothesis based on a top-down control of the insular cortex by supramodal midline networks is consistent with the results but should be considered with caution. Although the pI undoubtedly contains networks highly sensitive to thermal and/or painful events^58–61^, this cortex is also involved in the processing of other sensory inputs^62^, not only somatosensory but also vestibular and auditory, in particular related to temporal and spatial context analysis^63,64^. Since a fine-grained temporal dynamics of insular responses is not accessible to fMRI^30^, there are certainly temporal modulations of responses between posterior and anterior insular cortices, that could not be observed here^65^.

In conclusion, we show that empathetic and unempathetic comments appear to be integrated differently in high-level brain structures (i.e., PCC/Prec, vmPFC and DLPFC) generally involved in the processing of other’s attitudes, self-awareness and autobiographical recall. The subjective perception of pain intensity is the result of an interaction between different brain regions, and a number of networks appear to be tightly coupled. The increase of functional connectivity of the insular cortex in Empathetic condition may reflect a control process on this central region of pain processing, inducing a reappraisal of pain intensity perception. We show here that the recruitment of these brain areas by hearing empathetic feedback from others is involved in pain reduction. A better understanding of this neural system in the pain experience may allow us to identify new anatomical targets and non-pharmacologic methods for inducing effective pain relief.

## Material and Methods

### Participants

Thirty-six right-handed healthy subjects naive to the experiment participated to the study that was approved by the local Ethics Committee (CHU Saint-Étienne, Comité de Protection des Personnes, Sud-Est 1, France). They gave written informed consent according to the Declaration of Helsinki and received a compensation for their participation. Of them, 30 participants (16/30 males; mean age ± SD = 25.5 ± 6.0 years) completed the study. Six were excluded from analyses, because they either did not believe in the scenario (i.e., they considered the comments as not credible) or identified that the comments were a part of the experimental manipulation. Participants had no depression or anxiety according to the Beck questionnaire^66^ and the State-Trait Anxiety Inventory^67^, respectively. Cardiac abnormality, neurological, psychiatric, chronic pain or mood disorders were exclusion criteria; all subjects had similar socioeconomic and ethnic/cultural background.

### Experimental procedures and materials

The audio-scenarios and experimental setup were those validated in a previous psychophysical study^8^. The participants were unaware that this was an empathy-related experiment and were informed that the study’s goal was to collect brain data associated with their pain perception. The full aim of the study was revealed to them only at the end of the MRI session. Briefly, participants received noxious heat stimuli on their left hand and could inadvertently overhear discussions coming from the imaging staff, who commented on the participants’ attitude towards pain with different degrees of empathy. Subjects were scanned during three conditions (i.e., Neutral, Unempathetic and Empathetic), identical in terms of pain stimuli, but during which the degree of empathetic feedback expressed by the observers was changed. The neutral (N) comments were always heard at the beginning of the experiment, and concerned irrelevant discussion to make the participants familiar with the special situation of overhearing what should normally not be heard in an fMRI experiment (e.g., the conversation between the radiological staff outside the magnet room). Gradually, the conversations came to discuss the experiment, the volunteers, and their pain. However, keywords and sentences were selected to keep the neutrality of all comments^8^. This neutral exchange at the beginning of the experiment allowed a progressive and credible shift to distinct Empathetic/Unempathetic conversations. It also served as a control condition in the fMRI analysis. Then, the order of conditions with Empathetic (E) or Unempathetic (U) comments was counterbalanced across subjects (‘N-E-U’ or ‘N-U-E’) to avoid an order effect. The conversations were pre-recorded to ensure repeatability of the experiment. Participants and experimenters were introduced face-to-face to each other just before the test (without verbal exchanges), so that participants were able to attribute discussions to a group of persons they had previously met.

Each of the three experimental conditions consisted of two successive sessions, each lasting 8 min (Fig. 1a). The sessions were made of 12 thermal noxious stimuli alternated with 8 verbal stimuli. The term “verbal stimuli” (VS) refers to short verbal exchanges between experimenters (comments lasting 27.4 ± 14.3 s, range 10–50 s), interleaved with silences and background noise. Verbal stimuli were delivered through high-quality headsets (Nordic Neuro Lab fMRI audio system, Neuro Device, Poland) with a pseudo-randomized Inter-Stimulus Interval (mean ISI = 51.7 s ± 33.1 s). Thermal noxious stimuli were delivered through a 30 × 30 contact probe (Pathway Pain & Sensory Evaluation System, Medoc Ltd., Advanced Medical System, Israel) on the back of the left hand and alternated between a baseline temperature of 32 °C and a target noxious temperature determined individually for each participant just before the test^8^. Target temperatures were maintained for 10 seconds (+5 s of ascending and descending ramps) and were set in order to obtain a stable pain perception, rated around 60/100 by the participants (mean temperature of stimulation 46.8 ± 1.1 °C, range 44–49). Noxious stimuli were delivered during the silent interval between verbal stimuli, with a pseudo-randomized inter-stimulus interval (mean ISIp = 23.0 s ± 12.9 s) to avoid anticipation effects. The time period between the end of noxious stimuli and the onset of verbal stimuli was also variable and ranged from 12 to 20 seconds. A computer with E-Prime 2.0 software (Psychology Software Tools, Inc.) was used to ensure the synchronization of stimuli onset (noxious and verbal) and the online acquisition of pain ratings. At the end of the MRI acquisition, each volunteer was interviewed during a debriefing conversation with the aim to assess whether they had believed to the scenario and had perceived the different contexts. If they had, their data were considered for analysis. If not, they were excluded.

### Behavioral data

The volunteers rated pain intensity continuously by means of two response buttons (model Lumina LU400-PAIR; Cedrus Corporation, USA), one to decrease and the other to increase ratings on a VAS scale going from 0 (i.e., no pain) to 100 (i.e., worst imaginable pain). Rating was recorded in continuous which allowed to define a curve indicating pain intensity ratings (PR) as a function of time. In addition, for each noxious stimulus, the peak pain score (i.e., [PR]max) were assessed in order to test the effect of condition on pain ratings. Statistical analysis was performed using two-way, mixed design analysis of variance (ANOVA) with experimental conditions (N/E/U) as *within* factor, and the order of conditions (‘N-E-U’ or ‘N-U-E’) as *between* factor. Greenhouse-Geisser correction and Tukey post-hoc tests were applied following significant main effects or interactions (*p* < 0.05). Statistics were calculated with SPSS (Statistical Package for the Social Sciences, SPSS Statistics 20 Inc, Chicago USA).

### EPI images data acquisition, processing and analyses

Subjects were scanned in a 3T MR system (Prisma Siemens, Erlangen, Germany, EU) with a 64 channel head/neck coil. Structural T1-weighted images were acquired for anatomical reference during the first session of the Neutral condition with a 3D rapid gradient-echo sequence (MP-RAGE; TR = 2000 ms; TE = 2.24 ms, flip angle 8°, 192 sagittal slices, FOV = 230 mm, voxel size = 0.9 × 0.9 × 0.9 mm, matrix 256 × 256). Changes in blood oxygen level dependent (BOLD) T2* weighted MR signal were measured, using an interleaved gradient echo-planar imaging (EPI) sequence (TR = 2200 ms, TE = 30 ms, voxel size = 3.0 × 3.0 × 3.0 mm, flip angle 90°, slices/volume 40). A total of 220 EPI volumes were acquired in each functional session. The Statistical Parametric Mapping software (SPM12, Welcome Trust Centre for Neuroimaging, UK) was used for image processing and analyses. The first two scans of each session were removed, then images were slice-time corrected, spatially realigned, co-registered with the MNI152 brain using nonlinear warping, segmented using masks derived from the anatomical scan, and smoothed with a 6-mm FWHM Gaussian kernel. A high-pass temporal filter with a cutoff of 128 s was then applied.

#### First-level individual analyses

Multiple brain regions are activated by a nociceptive stimuli and reflect different processes leading to the final pain perception^9,10,12,25^. Most previous neuroimaging studies on pain did not distinguish brain responses related to the sensory representation of the nociceptive stimuli from those corresponding to the conscious perception of pain intensity. Recording continuous ratings of pain perception during the experiment provides a solution to separate these distinct activations^28^. Here, the pain stimulation was split into two periods (Fig. 1a) according to the onset of pain intensity ratings by the subject. The aim was to capture specifically the brain activity correlated to the magnitude of pain ratings, and to assess the effect of Empathetic condition on it.

A within-subject general linear model (GLM) design matrix was built and incorporated 5 functional sessions corresponding to experimental conditions; each included three regressors of interest (see Fig. 1a). The first regressor described Verbal Stimuli [VS], i.e., the short phrases with different empathetic value. The second regressor captured the brain activity corresponding to the period between the onset of thermal noxious stimulation and the onset of pain perception ratings, called “pre-pain” [prePAIN] i.e., the beginning of the nociceptive stimulation that was not yet judged as painful by the subjects. The third regressor, successive to the second, described the window where pain perception ratings [PR] were produced; i.e., the time period when the subjects felt the stimulus as painful (pain ratings ≠0) until they felt no pain and came back the scale’s cursor to 0. [prePAIN] and [PR] regressors were orthogonalized to minimize the occurrence of collinearity in the model^68^, and convolved with the canonical hemodynamic response function (hrf) and its time-derivative. Six time-series of head movement parameters were added in the linear model as covariates of no interest to remove residual variance.

#### Second-level group effect analyses

Group analyses were performed to assess (1) the brain activity related to regressors for all stimuli delivered during the experiment, whatever the conditions (Fig. 1b), in order to show the brain network associated with them compared to the baseline (thresholded at *p* < 0.01 FWE corrected voxel level); and (2) the effects of the experimental conditions (N, E, U) on the behavioural pain scores [PRmax] and on the brain activity related to conscious pain perception [PR]. These BOLD contrasts between conditions aim to reveal the neural activations and deactivations associated with the effect of Empathetic and Unempathetic conditions controlled for the Neutral condition during pain assessment. For each voxel, experimental conditions were compared using paired sample t-tests. The resulting SPM {z} maps were thresholded at *p* < 0.001 and clusters that survived FWE correction for multiple comparisons *p* < 0.05 were reported, revealing the brain activities associated with empathetic and unempathetic feedback effects on pain perception. Anatomical regions were labeled using both the Hammersmith^69,70^ and the Human Connectome Project atlases (HCP MMP 1.0)^71^.

### Functional connectivity (FC) analyses

Connectivity analysis aimed at investigated the between-areas relationships (i.e., BOLD signal synchrony) modulated by experimental conditions. Based on previous literature, we selected a subset of brain regions that could plausibly mediate the effect on pain perception, from the set of brain regions (i.e., main clusters) that were significantly activated in the task analysis. We utilized that subset to define volumes of interest (VOI) for the connectivity analysis. These brain regions represented a short part of the whole functional network involved but focused on the main areas that specifically contributed to our effect. VOI were defined on the basis of their activation peaks and corresponding HCP brain atlas^71^. Figure 3a depicts these VOI.

#### Time-series extraction, correlation matrices, weighted signed modularity

FC analysis design was achieved under the open-source python package nipype^72^. The Neuropycon project was used for extracting time-series over VOI and computing FC matrices. Time-series averaged over the voxels within the VOI were extracted for each of the participants. Head movement parameters (rotation and translation), as well as average signals in the white matter and CSF were regressed out from VOI time series, and the residuals of the regression were kept. Residuals were then high-pass filtered (>0.01 Hz) to remove the scanner drift component of the signal and normalized (Z-score) for each experimental condition.

#### Task-based FC

We computed FC relative to pain stimulation using weighted correlations for each experimental condition^73^. This corresponds to computing Pearson correlation between two time-series over the whole session, weighting the contributions of each time points by the pain stimuli, giving more influence to periods where the regressor is high, and lower influence when the regressor is low or null. The pain stimulation was convolved with a canonical hrf and only the positive and null parts of the hrf were used to compute weighted correlations of VOI structure time-series pairs. This resulted in a set of correlation matrices (14 × 14) for each condition.

#### FC statistic comparisons

Weighted correlation matrices of each experimental condition were compared with paired sample T tests. A false positive correction for multiple comparisons, limiting the risk of type II errors, was retained, because it has been demonstrated to be a suitable threshold in order to determine the significance of connectivity results^74^.

## Supplementary information

Supplementary information: brain activations tables and figure 4

## Data Availability

The datasets generated during and/or analysed during the current study are available from the corresponding author on reasonable request.
