# Supplementary material for: Brain activity sustaining the modulation of pain by empathetic comments
Source: Sci Rep. 2019 Jun 10;9:8398. doi: 10.1038/s41598-019-44879-9 (PMC6558033; doi:10.1038/s41598-019-44879-9)
Supplement: Supplementary file 1 — Supplementary information: brain activations tables and figure 4 [file 41598_2019_44879_MOESM1_ESM.pdf]

Brain activity sustaining the modulation of pain by empathetic comments

(Original article)

*C. Fauchon[(a)], I. Faillenot[(a)], C. Quesada[(a)], D. Meunier[(a, c)], F. Chouchou[(a)], L. Garcia-Larrea[(a)], R. Peyron[(a, b)]

(a) Central Integration of Pain, Lyon Neuroscience Research Center, Inserm U1028, UCB Lyon1 & UJM, F-42023 Saint-Etienne, France

(b) Department of Neurology & Pain Center, CHU de Saint-Etienne, F-42055 Saint-Etienne, France

(c) Aix Marseille Univ, CNRS, INT, Inst Neurosci Timone, Marseille, France

*Corresponding author: Camille Fauchon, Inserm U1028, Intégration Centrale de la Douleur, Faculté de Médecine J.Lisfranc, 10 rue de la Marandière, F-42 270 Saint-Etienne cedex 2, France.

Phone : +33 477 127 805; Fax: +33 477 120 543;

Email : **camille.fauchon63@gmail.com**

Supplementary material

Table 1: Main cortical peak activations for [VS], [prePAIN] and [PR] regressors independently of the empathetic conditions (see Fig. 1.). (Voxel level *p* (FWE corr) < 0.01 and cluster size > 57 voxel (resel))

[Regressor] cluster size	Side	localization Macro-anatomical Hammers's atlas (HCP regions or Morel areas)	Peak coordinates MNI(x , y, z)	Z score
**[VS]**				
880	L	Superior Temporal Gyrus, middle part (TA2, A1)	-51 -13 2	7.56
		Middle Temporal Gyrus (STSdp)	-51 -25 -4	7.10
		STG, anterior part (STGa)	-48 14 -13	5.94
		Inferior Frontal Gyrus (IFSp, 47L, 45)	-54 26 11	6.73
563	R	Superior Temporal Gyrus, middle part (A4, STSda, A1, 52, STSdp, Lbelt)	60 -13 -1	7.81
498	L/R	Superior Frontal Gyrus (9m, 8BL)	-9 50 23	7.81
134	L/R	Superior Parietal Gyrus (d23ab, 31a)	3 -55 23	6.19
	L/R	Posterior Cingulate Gyrus (d23ab, v32ab)	5 -46 29	5.63
**[prePAIN]**				
3468	L/R	Post-mid cingulate gyrus (a24pr, 33pr)	-3 11 32	7.52
	L/R	sup frontal gyrus (SCEF, 6ma)	-5 5 53	7.10
	L	precentral gyrus (6d, FOP1)	-39 -10 59	7.20
	L/R	anterior insula (FOP3, FOP4, MI, FEF)	-33 11 8	7.77
	L	parietal operculum (OP1, OP4)	-60 -19 23	7.63
	L	posterior insula short gyrus (PoI2, Ig)	-37 -1 8	7.39
	L	pallidum	-18 -1 -1	7.49
	L	putamen	-24 5 -1	7.21
1652	R	anterior insula (MI)	39 17 -1	7.63
		parietal operculum (OP1, OP4)	57 -16 16	6.74
		posterior insula cortex (PoI2, Ig)	36 5 -10	6.64
		frontal gyrus (FOP4, 44, FOP5)	48 5 5	7.84
		putamen	21 14 -4	7.13
		thalamus (VL, LP, VPL, CM, PuA, VP)	12 -13 5	7.05
157	L	Dorsolateral prefrontal cortex (9-46d, 46, 9-46v)	-30 41 29	7.42
288	R	Dorsolateral prefrontal cortex (46, 9-46v, 9-46d)	33 41 29	7.13
171	L/R	postcentral gyrus (RSC, 23c, 5mv)	6 -13 32	6.56
**[PR]**				
483	L/R	Anterior insula short gyrus (MI, FOP5, AVI)	34 17 1	6.42
175	R	Dorsolateral prefrontal cortex (44, 46, 9a)	36 47 29	6.40
180	L	Inferior frontal gyrus (FOP4)	-33 14 11	6.31
155	R	Ant-mid cingulate gyrus (a24pr, 8BM, SCEF)	9 23 47	6.21
		Superior frontal gyrus (SCEF)	5 17 32	6.21

Table 2: Main cortical peak activations related to experimental conditions comparison (Paired t-tests corrected: Empathetic vs Unempathetic vs Neutral) during pain perception assessment [PR]. Statistical maps are thresholded at FWE-corrected cluster-based $p < 0.05$ after voxel threshold at $p < 0.001$.

[Regressor] cluster size (pFWE-corr)	Side	localisation Macro-anatomical Hammers's atlas (HCP regions or Morel areas)	Peak coordinates MNI(x , y, z)	Z score
[E>U]				
111	L/R	Precuneus (7m)	-6 -55 35	4.86
(0.002)	L/R	posterior cingulate gyrus (31a, 31pv)	14 -46 41	3.60
[E>Neutral]				
379	R	Anterior insula short gyrus (MI, AVI, 44, 6r)	51 8 14	5.01
(0.000)				
124	R	Posterior parietal cortex	54 -34 47	4.95
(0.001)		(PF, AIP, 7PC, Pfop, OP4, PFt, 1, 2)		
73	R	Dorsolateral prefrontal cortex (a9-46v)	39 44 20	3.55
(0.016)				
[E< Neutral]				
85	L	middle frontal gyrus (8Av, 8Ad)	-30 24 53	3.60
(0.008)				
[U> Neutral]				
86	R	Posterior parietal cortex (PF)	54 -34 50	4.32
(0.007)				
66	R	Insula: anterior short gyrus (AVI, AAIC	30 23 8	4.22
(0.022)		FOP4)		
[U< Neutral]				
2100	L	angular gyrus (PGp, PGs, PGi)	-42 -73 26	5,63
(0.000)	L/R	posterior cingulate gyrus/Precuneus	-12 -40 35	4.52
	R	precentral gyrus (6d, 4)	33 -16 65	4.49
286	L/R	subgenual frontal cortex (s32, 25, 10v)	9 29 -13	5.06
(0,000)	L/R	superior frontal gyrus (10r, 9m)	-3 56 -7	4.31
301	R	angular gyrus (PGi, PGs)	45 -55 23	4.91
(0,000)				
276/394	L/R	hippocampus (H, PHA2, PHA3, Pres, TGd)	26 -19 -19	4.49
(0.000)				
224	L	superior/middle frontal gyrus (8BL, 8Ad)	-23 23 42	4.19
(0.000)				

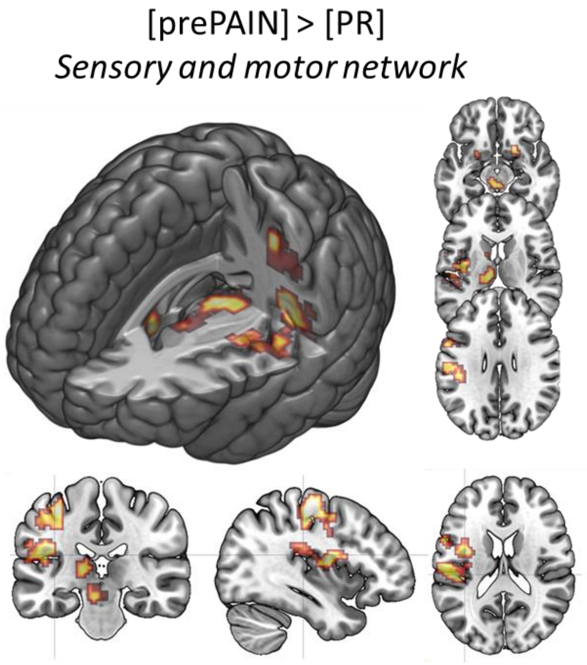

[Regressor] cluster size	Side	localization Macro-anatomical Hammers's atlas (HCP regions or Morel areas)	Peak coordinates MNI(x , y, z)	Z score
**[prePAIN] > [VRS]**				
316	L	Parietal operculum (OP1-4)	-42 -26 24	4.62
		Posterior insula (Ig, Pol2)	-38 -12 10	4.20
281	L	Gyrus postcentral (3b) / precentral (4)	-33 -24 56	5.14
301/103	L/R	Thalamus	-6 -19 11	4.31
		Putamen	-18 14 -10	4.89
		Brainstem	-6 -24 -16	3.61

Figure 4. Brain activity related to noxious stimulus reception. Split the pain experience in two acts also allow an access to the BOLD activity related to the reception of the nociceptive stimuli, i.e. the first cortical region recruited by a noxious stimulus. The ontrast between the [prePAIN] and [PR] regressors ([prePAIN] > [PR]), with inclusive mask on [prePAIN] activation map) represented the activations related to the engagement of motor and sensory brain network in the first brain reception steps of noxious signal. The largest clusters activations (table above) were found bilaterally in the thalamus (including putamen) and brainstem, in the left: parietal operculum (OP1 and OP4), dorsal posterior insular cortex (IG and Pol2), postcentral and precentral gyrus. Statistical maps are thresholded at FWE-corrected cluster-based $p < 0.05$ after voxel threshold at $p < 0.001$.
